# COVID-19 research in South Asia: a bibliometric analysis of the 100 most-cited articles

**DOI:** 10.3205/dgkh000448

**Published:** 2023-09-21

**Authors:** Bisal Naseer, Mohsan Ali, Neha Azhar

**Affiliations:** 1 King Edward Medical University, Lahore, Pakistan; 2 Khawaja Muhammad Safdar Medical College, Sialkot, Pakistan

**Keywords:** COVID-19, coronavirus, bibliometric analysis, citation, South Asia, Pakistan, Islamic Republic, Bangladesh, Bhutan, India, Nepal, Sri Lanka, Afghanistan, Pemba Islands, Maldives

## Abstract

**Background:**

With the surge in the number of infected individuals during the COVID-19 pandemic, there was also a surge observed in the number of publications discussing its epidemiology, characteristics, path-o-phys-i-ol-o-gy, diagnosis, prevention and treatment. This bibliometric analysis focuses on the papers published on COVID-19 in South Asia.

**Methods:**

We searched articles in the Scopus database from December 2019 to October, 2022. After manual screening, a list of the 100 most-cited articles was obtained, which was analyzed for various factors, including the type of article, citation count, author’s affiliation, country of origin, funding bodies, etc.

**Results:**

The majority of the top 100 articles (n=79) in South Asia were published during 2020. India was affiliated with the highest number of articles (n=68), followed by Bangladesh (n=18) and Pakistan (n=12). However, 7 articles were authored by a researcher in Bangladesh. Female authors were under represented (32.38%), with no female author in lists of authors with 4 or more articles. The average number of citations for each of the top 100 most-cited articles was 180.8. Original articles constituted the major portion of the publications (82%), followed by letters (11%) and reviews (4%). Half of the publications belonged to the field of medicine (n=49), while others were contributed by science, psychology, social sciences, and biochemistry and allied sciences (n=8). Vaccine trials were under-represented. Jahangirnagar University, Bangladesh was affiliated with the maximum number of articles. Most articles were published in Science of The Total Environment (n=8) while Indian Council of Medical Research (n=4) was the top funding body.

**Conclusion:**

These findings highlight that South Asia has a great potential to conduct research addressing its challenging health problems. But lack of funds hinders conducting trials of new medications and vaccines. Thus, there is need for allocation of sufficient funds for research and clinical trials by governments and the private sector to enhance the research productivity of this region.

## Zusammenfassung

**Hintergrund:**

Mit dem sprunghaften Anstieg der Zahl von COVID-19-Infektionen stieg auch die Zahl der Veröffentlichungen, die sich mit der Epidemiologie, Merkmalen, Pathophysiologie, Diagnose, Prävention und Behandlung der Erkrankung befassen. Die bibliometrische Analyse konzentriert sich auf die in Südasien veröffentlichten Arbeiten über COVID-19.

**Methode:**

Von Dezember 2019 bis Oktober 2022 wurden Artikel in der Scopus-Datenbank recherchiert. Nach manuellem Screening wurde eine Liste der 100 meist zitierten Artikel erstellt, die auf verschiedene Faktoren analysiert wurde, darunter die Art des Artikels, die Anzahl der Zitate, die Zugehörigkeit des Autors, das Herkunftsland, die Finanzierung usw.

**Ergebnisse:**

Die meisten der 100 wichtigsten Artikel (n=79) wurden im Jahr 2020 veröffentlicht, davon die Mehrzahl in Indien (n=68), gefolgt von Bangladesch (n=18) und Pakistan (n=12). Die meisten der Artikel (n=7) wurden von einem Forscher aus Bangladesch verfasst. Weibliche Autoren waren unterrepräsentiert (32,38%); keine Autorin war in der Liste der Autoren mit 4 oder mehr Artikeln vertreten. Die durchschnittliche Anzahl der Zitate für jeden der 100 am häufigsten zitierten Artikel betrug 180,8. Der größte Teil der Veröffentlichungen waren Originalartikel (82%), gefolgt von Leserbriefen (11%) und Rezensionen (4%). Die Hälfte der Veröffentlichungen stammte aus dem Bereich der Medizin (n=49), die anderen aus den Naturwissenschaften, der Psychologie, den Sozialwissenschaften sowie der Biochemie und verwandten Wissenschaften (n=8). Impfstoffstudien waren unterrepräsentiert. Die meisten Artikel kamen aus der Jahangirnagar-Universität in Bangladesch. Acht Artikel wurden in Science of the Total Environment (n=8) veröffentlicht; der Indian Council of Medical Research (n=4) war der wichtigste Geldgeber.

**Schlussfolgerungen:**

Die Ergebnisse zeigen, dass Südasien ein großes Potenzial für die Durchführung von Forschungsarbeiten zur Lösung seiner schwierigen Gesundheitsprobleme hat. Jedoch behindern fehlende Budgets die Durchführung von Untersuchungen mit neuen Medikamenten und Impfstoffen. Daher müssen ausreichende Mittel für Forschung und klinische Studien auf staatlicher und privater Ebene bereitgestellt werden, um die Produktivität der Forschung in dieser Region zu steigern.

## Introduction

Initially, it was believed that the COVID-19 virus only affects the lungs, and results in asymptomatic or mild respiratory infections to severe respiratory distress [1], [2], [3] [4]. By now, however, studies have proved that it also affects other organ systems, including the brain, heart, kidneys, liver, skeletal muscles, and skin [5]. The initial cases of novel coronavirus pneumonia that occurred in Wuhan, China in December 2019 [6] were followed by a global pandemic [7] with cases reported in more than 213 countries. According to the WHO, as of 18 July, 2023, 768 million confirmed cases and 6.95 million deaths have been reported globally [8]. The deadly pandemic struck South Asia on 23^rd^ January 2020 [9], when Nepal reported its first case of a man who had recently traveled to Wuhan. Up to now, there have been 50.36 million confirmed cases and over 600,000 deaths reported for South Asia, with India having the greatest number of deaths [10], [11].

With the emergence of this deadly pandemic, the scientific community all over the world responded by investigating the epidemiology, characteristics, pathophysiology, diagnosis, and prevention of this viral disease. This led to an enormous amount of research, with the USA, UK, China, and Italy being leading nations contributing to COVID-19-related research [12], [13]. Since three South Asian countries. i.e., India [14], Pakistan, and Bangladesh outnumbered China in COVID-19 cases [15], it becomes very crucial to evaluate the research performance of this region in the COVID-19 pandemic era in pursuit of a timely response. Bibliometric quantitative analysis of journal articles and their accompanying citation counts were performed for this purpose [16]. The basic tool in bibliometrics is citation analysis, which involves citation count: the number of times a source item is cited [17]. This method not only helps to find the impact of articles in a specific field [18], but also directs those in government offices, funding agencies, labs, and the research directors to decide to support a research topic based on recent trends and advances in each field. 

Although there have been bibliometric studies on COVID-19-related research trends on a global scale [19], [20], [21], also focusing on Africa [22], [23] and the Arab world [24], the research framework of COVID-19 in South Asia and its research performance during this pandemic have been so far neglected. Thus, this bibliometric study aims to describe the characteristics of the 100 most-cited articles among COVID-19 publications from this region in terms of their country of origin, level of evidence, journal of publication, research themes, local author’s contributions, affiliated institutes, and funding bodies. This will not only highlight the hot spots and the areas that are neglected in this region, but also the capacity for local research and its lack, so that effective planning and funding can be provided for future research.

## Method

### Data source and study selection

The search in Scopus was restricted to articles published in English up to October 16, 2022. All articles with a focus on COVID-19 in South Asia (either as a whole or any country in South Asia) were included. Those which included any other country than Afghanistan, Bangladesh, Bhutan, India, Maldives, Nepal, Pakistan, and Sri Lanka were excluded. No restriction regarding the kind of article was applied.

### Search strategy

Keywords pertaining to South Asia, individual countries in this region, and Covid-19 were obtained from PubMed. These keywords were later structured into a detailed search strategy as follows:

**TITLE-ABS-KEY (**covid 19**) OR (**covid-19 **AND** pandemic**)** **OR** **(**sars-cov-2 **AND** infection**)** **OR** **(**coronavirus **AND** disease 19**) OR** **(**sars **AND** coronavirus 2 infection**)** **OR** **(**coronavirus **AND** infection**)** **OR** **(**covid19**)** **OR** **(**wuhan **AND** virus**)** **OR** **(**severe **AND** acute **AND **respiratory **AND** syndrome **AND** coronavirus 2 infection**) OR** **(**2019 novel **AND** coronavirus **AND** disease**)** **OR** **(**novel **AND** cov**)** **OR (**sarscov2**)** **OR** **(**2019ncov**)** **OR (SARS-CoV-2 OR COVID-19 linked to Islamic** republic, **Bangladesh, Bhutan, India**, Nepal, **Sri Lanka**, south **Asia, Afghanistan**, pemba, cocos islands **OR** Maldives.

The search words were used in the title/abstract/keyword field of Scopus. Later, the results were arranged according to the highest citation (in descending order), and manual screening was conducted until the 100 most-cited relevant articles were obtained. Any articles that did not focus solely on Covid-19 in South Asia were excluded.

### Analysis

The selected studies were analyzed by Scopus itself and yielded tables for authors, authors’ affiliations, countries of origin, type of manuscript, funding sponsor, journal, subject, and year of publication. The rest of the analyses were conducted on Numbers, version 12.1 (7034.0.86), by Apple Inc.

## Results

The total number of COVID-19 related publications obtained by applying the above-mentioned search terms in the Scopus search engine was 14,914. Out of these. We included only the 100 most-cited articles matching our inclusion criteria.

### Year-wise analysis

**Figure 1:**
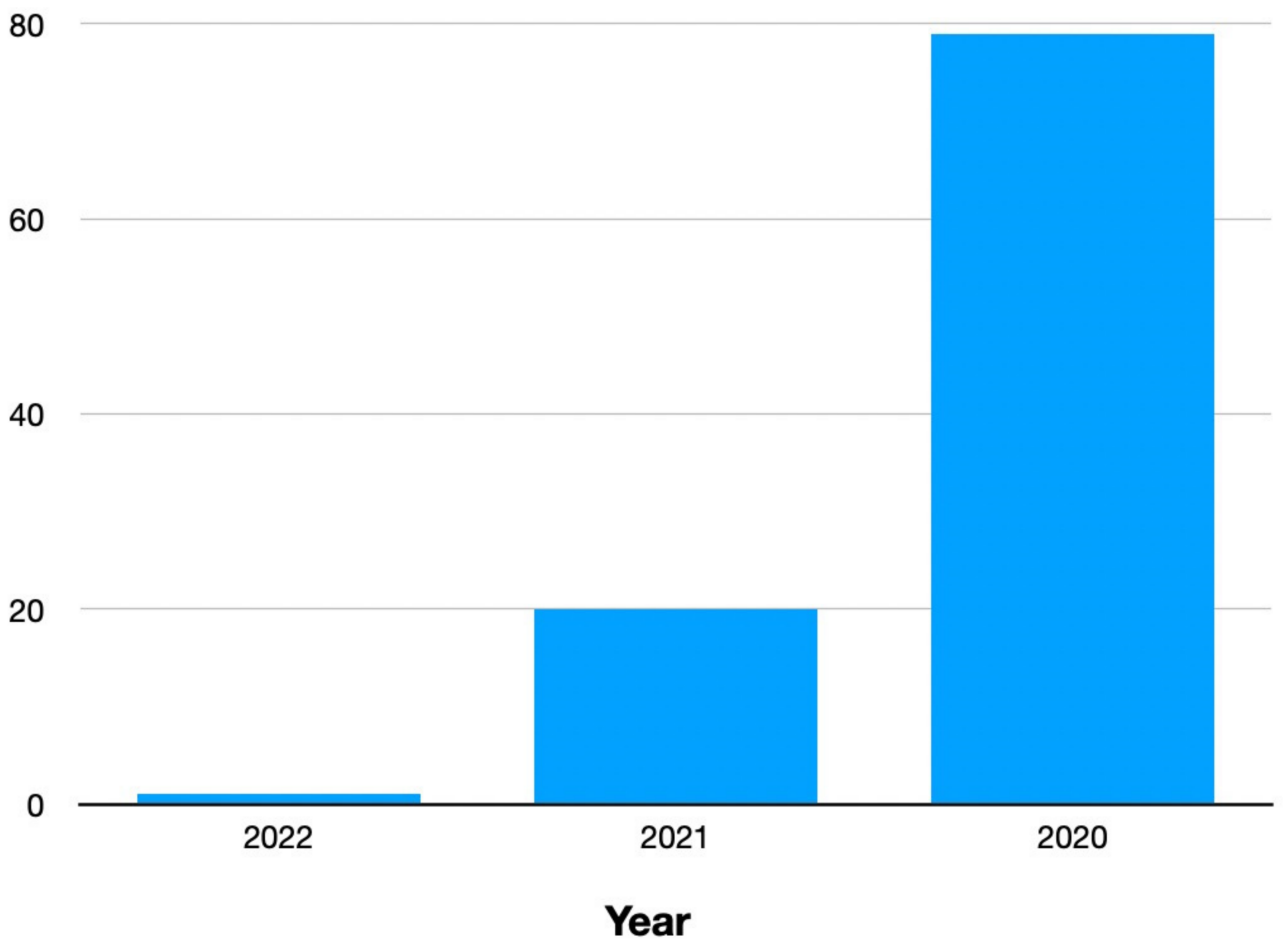
Number of articles published each year

Figure 1 (Fig. 1) shows that out of a total of 100 papers, 79 were published in 2020, and 20 papers were published in 2021 that focused on COVID-19, solely in South Asia. On the other hand, the year 2022 had only one single published paper. 

### Authors

A total of 1,312 authors contributed to the 100 most-cited articles related to COVID-19 research in South Asia. An average number of 13.1 authors was found for one article. Out of these, 734 (55.9%) authors were identified to be affiliated with a South Asian Institute. 67.7% (n=497) of authors from this region were male; female authors made up 32.28% (n=237). Authors having 4 or more articles in the 100 most-cited articles are listed in Table 1 (Tab. 1). 

### Performance by country

Out of 8 South Asian countries, only 4 were presented as the setting among the 100 most-cited articles. India was the country with the highest number of country-focused articles (n=68). Bangladesh was the study setting in 18 articles, Pakistan in 12, and Nepal in 3 (Table 2 (Tab. 2)). 

### Citation analysis

The study entitled “Study of knowledge, attitude, anxiety & perceived mental healthcare need in Indian population during COVID-19 pandemic” [25] had the highest number of citations, i.e., 833. An article published in Nature entitled “SARS-CoV-2 B.1.617.2 Delta variant replication and immune evasion” [26] received the maximum number of citations per year, i.e., 447. The average number of citations for each of the top 100 most-cited articles was 180.8, and the median number of citations was 144. However, the median of citations per year (CPY) was 88. The average of citations per year for 2020, 2021, and 2022 is given in Table 3 (Tab. 3). 

### Affiliated institutes, funding bodies and journals

A list of institutes affiliated with 4 or more articles is given below in Table 4 (Tab. 4). The institutes that were at the forefront in the field of COVID-19-focused research in South Asia were Jahangirnagar University, Bangladesh (n=13), Indian Council of Medical Research (n=8), CHINTA research, Bangladesh (n=7) and All India Institute of Medical Sciences (n=6). The Indian Council of Medical Research was the body funding the greatest number of articles (n=4), followed by other funding bodies from India, China, and the USA. A list of the top 15 funding bodies is given below in Table 5 (Tab. 5). The top 10 journals with maximum number of articles are ranked in Table 6 (Tab. 6). 

### Type of articles

While analyzing the data further (Table 7 (Tab. 7)), it was noted that original articles constituted the major portion of the most cited publications (82%), followed by letters (11%) and reviews (4%). The remaining 3% of the publications were conference papers, editorials, and notes, with 1% each. 

### Subject-wise analysis

**Figure 2:**
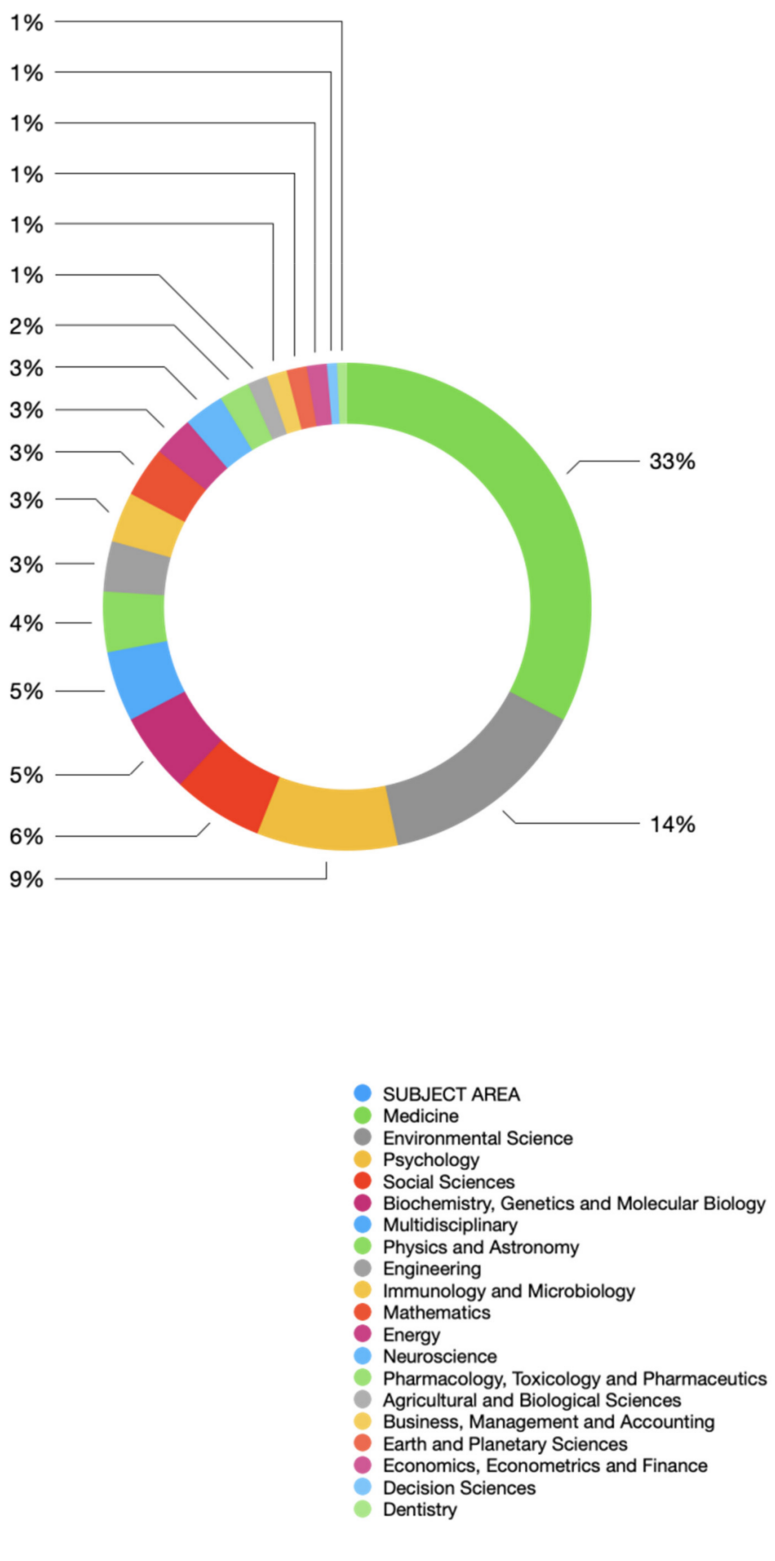
Subject-wise analysis

Figure 2 (Fig. 2) shows that half of the publications belonged to the field of medicine (n=49), while significant numbers were also contributed by environmental science (n=21), psychology (n=14), social sciences (n=9), and biochemistry, genetics and molecular biology (n=8). Seven papers were multidisciplinary, while 6 were from physics and astronomy. Engineering, immunology and microbiology, and mathematics had 5 papers each. Energy and neuroscience had 4 papers each. Three publications were from pharmacology, toxicology and pharmaceutics. Agricultural and biological sciences, business and management, earth and planetary sciences, and economics and finance showed a contribution of 2 publications per each subject. The least contribution, with 1 publication per subject, was made by decision sciences and dentistry. Only 4 randomized controlled trials (vaccine and treatment trials) were identified.

## Discussion

The COVID-19 pandemic has not only brought immeasurable suffering, but undoubtedly led to a massive research output, research collaborations and scholarly communication on a global scale. This was no different for South Asia.

This study shows that 2020 was the year that saw a sudden surge in publications and citations. In the context of Covid-19, it may be in part due to changes in the journal standards and the increased need for research in the face of an expanding pandemic [27]. In contrast, the years 2021 and 2022 showed a decline in the number of citations, perhaps because of revised protocols by the publishing journals as the need for more stringent scientific guidelines emerged. Surprisingly, despite the research circumstances during the pandemic, most of the papers were original articles rather than letters, reviews, and other forms of publications. A reason for this may be, as stated above, leniency and greater acceptance of publications with quality below par [28]. Another reason may be the increased availability of open-access articles on the major research platforms [29]. Despite this promising output, we found only 4 clinical trials among top 100 articles. This number is considerable lower that the number of clinical trials carried out in regions like China, Europe and America [30]. This might be due to lack of local funding and insufficient investment in research and health care in this region. Therefore, it is of utmost importance that the South Asian countries conduct randomized control trials to generate evidence relevant to the local population. We also argue that there is a dire need to conduct further research on safe vaccine development, treatment and prevention of COVID-19 in this region to mitigate its burden. 

Further evaluation of the origin and settings of the articles revealed a wide gap between the South Asian countries in the context of their contribution to the body of research related to COVID-19. We found that India led all the South Asian countries. This came as no surprise, as the scale of research and scientific advancement in India was already receiving massive support from its copious funding sources and the structure of its research institutes [31]. During the COVID-19 pandemic, a tremendous amount of scientific research specifically in the context of vaccine development was being carried out in India [32] Four of the top funding bodies in this region are based in India. This availability of local funding is an important factor for increased research output from India. Lastly, India has the highest GDP in South Asia, which might also account for this research productivity [33]. Other South Asian countries, namely Bangladesh, Pakistan, and Nepal, despite limited resources and funding, contributed a significant amount to the literature. The single institution contributing to a maximum number of research articles [n=13] is Jahangirnagar University from Bangladesh. This contribution to research is appreciable, as worldwide restrictions and a halt in economic growth posed great challenges during the pandemic [34]. Afghanistan, Bhutan, Sri Lanka and Maldives were not represented among the 100 most-cited articles. Many factors are responsible for this; the low gross domestic product (GDP) of these countries is a hindrance to their research performance. On average, South Asian countries spend less than 1% (0.64%) of their GDP on research and experimental development [35], which is quite low on the global scale. Moreover, outbreaks of infectious diseases like TB, AIDS, malaria, typhoid, dengue and hepatitis have overburdened already compromised healthcare systems in this region [36], [37].

Analysis of the authors of the 100 most-cited articles indicates that 55.9% (n=734) of them were based in South Asia. The top 3 authors’ affiliations were also South Asian institutions. This is promising, as it indicates the ability of local researchers and scientists to update the pool of knowledge on prevalent health problems for their efficient control and prevention. 54.1% (n=578) non-South Asian authors participated in research conducted in this region in collaboration with South Asian authors. Collaboration is essential to scientific creativity, because it makes it easier to share ideas and widens the variety of viewpoints on a particular topic [38]. International collaboration can produce substantial benefits for nations with lower GDP by allowing them to utilize the resources of nations with more developed research systems [39]. Hence, the number of collaborations with international authors must be encouraged to keep pace with global demands. Likewise, intra-regional collaborations can help South Asian countries address common regional health challenges. Female authors had a representation of only 32.38% (n=237), with none of them among top cited authors. Only one in three scientists (33%) is a woman, according to the UNESCO Science Report 2021. This gender disparity in research needs to be eliminated by providing equal funding and research opportunities to female researchers.

Most of the articles were published in Science of the Total Environment (n=8) and other international journals. Only 3 local journals, i.e. Asian Journal of Psychiatry, Indian Journal of Medical Research and Indian Journal of Ophthalmology, made the list of top 10 journals. This highlights the need for the availability of more local journals where South Asian authors can publish their work and which can address local concerns.

As expected, most of the articles were from the field of medicine, as scientists were working to uncover new facts and make discoveries related to COVID-19. This was the need of the hour because of the morbidity and mortality associated with this disease and the evolving nature of clinical presentation along with new emerging virus variants [40]. Moreover, increasing trends of digitization led to a shift in clinical studies towards electronic and virtual approaches [41]. Nevertheless, scientific research in other fields also continued and contributed to a more integrated pool of scientific knowledge [42].

## Conclusion

This bibliometric analysis highlights the COVID-19 research conducted in South Asia in terms of the 100 most-cited articles. A majority of the authors and their affiliated institutes are based in South Asia, which indicates satisfactory performance of this region to global research conducted on COVID-19. However, research participation is uneven in this continent, with 3 countries, i.e., India, Pakistan and Bangladesh, contributing the major share with little representation from other countries. This signifies the need for international collaborations, support and funding by South Asian governments for local research development.

## Notes

### Competing interests

The authors declare that they have no competing interests.

### Availability of data and materials

All data generated or analyzed during the study are included in this published article [and its supplementary information files].

### Funding

No funding received.

### Authors’ contributions

BN conceived of the research topic. BN and MA together conducted literature search. They screened articles individually on Scopus and matched their included studies to resolve any dis-cor-dance. MA performed descriptive analysis. BN, MA and NA wrote the draft. MA and BN read and approved the final manuscript.

### Author’s ORCHID

Bilal Naseer: 0000-0002-9713-1255

## Figures and Tables

**Table 1:**
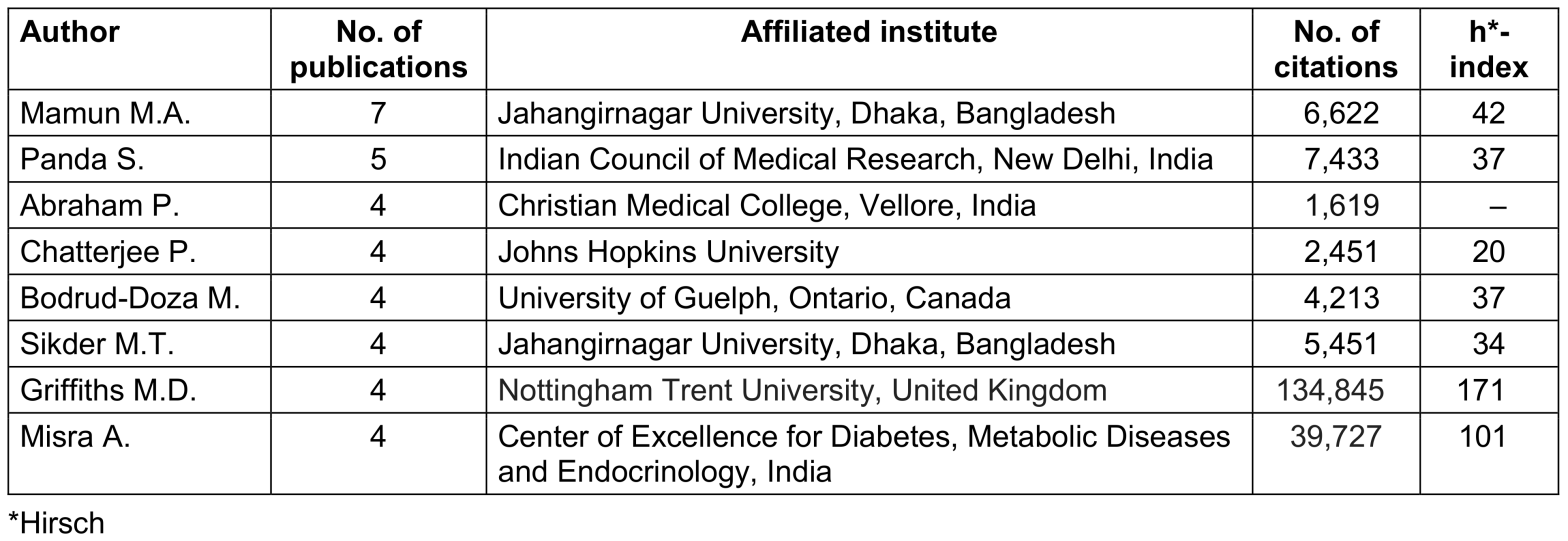
Authors with 4 or more articles in the top 100 list

**Table 2:**
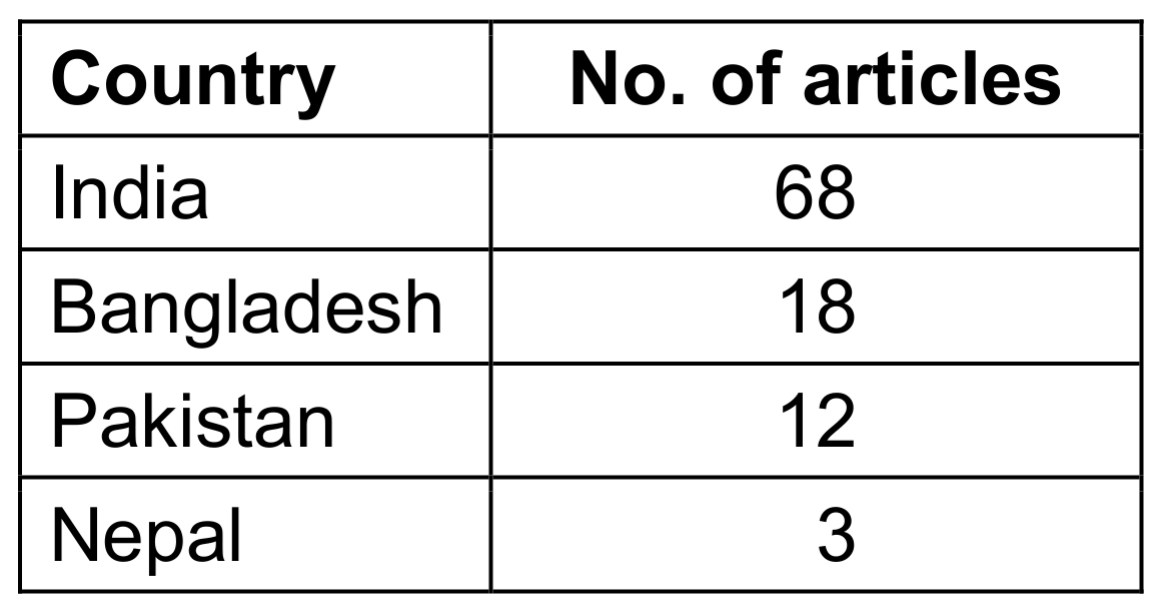
Top study settings

**Table 3:**
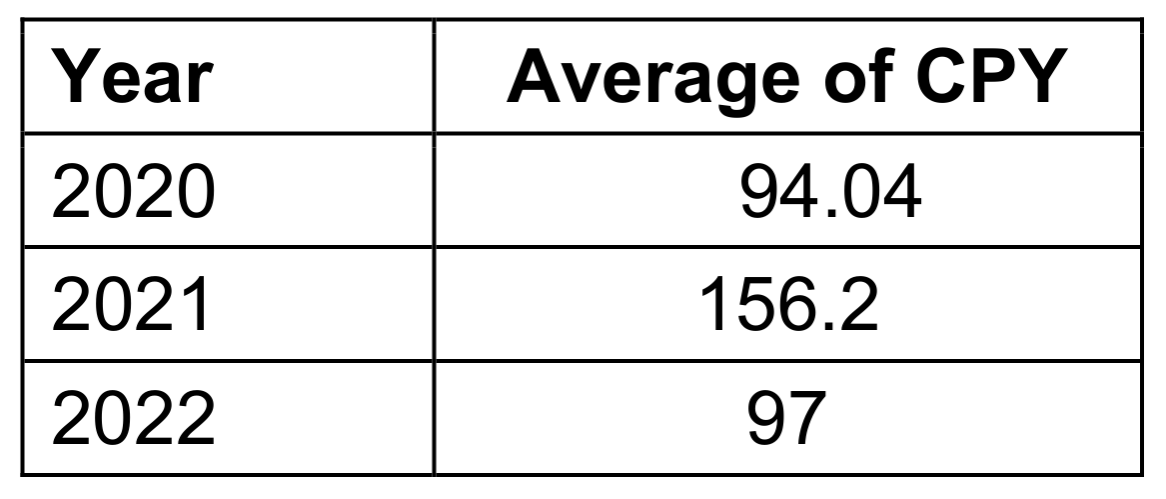
Average number of citations per year (CPY) in each year

**Table 4:**
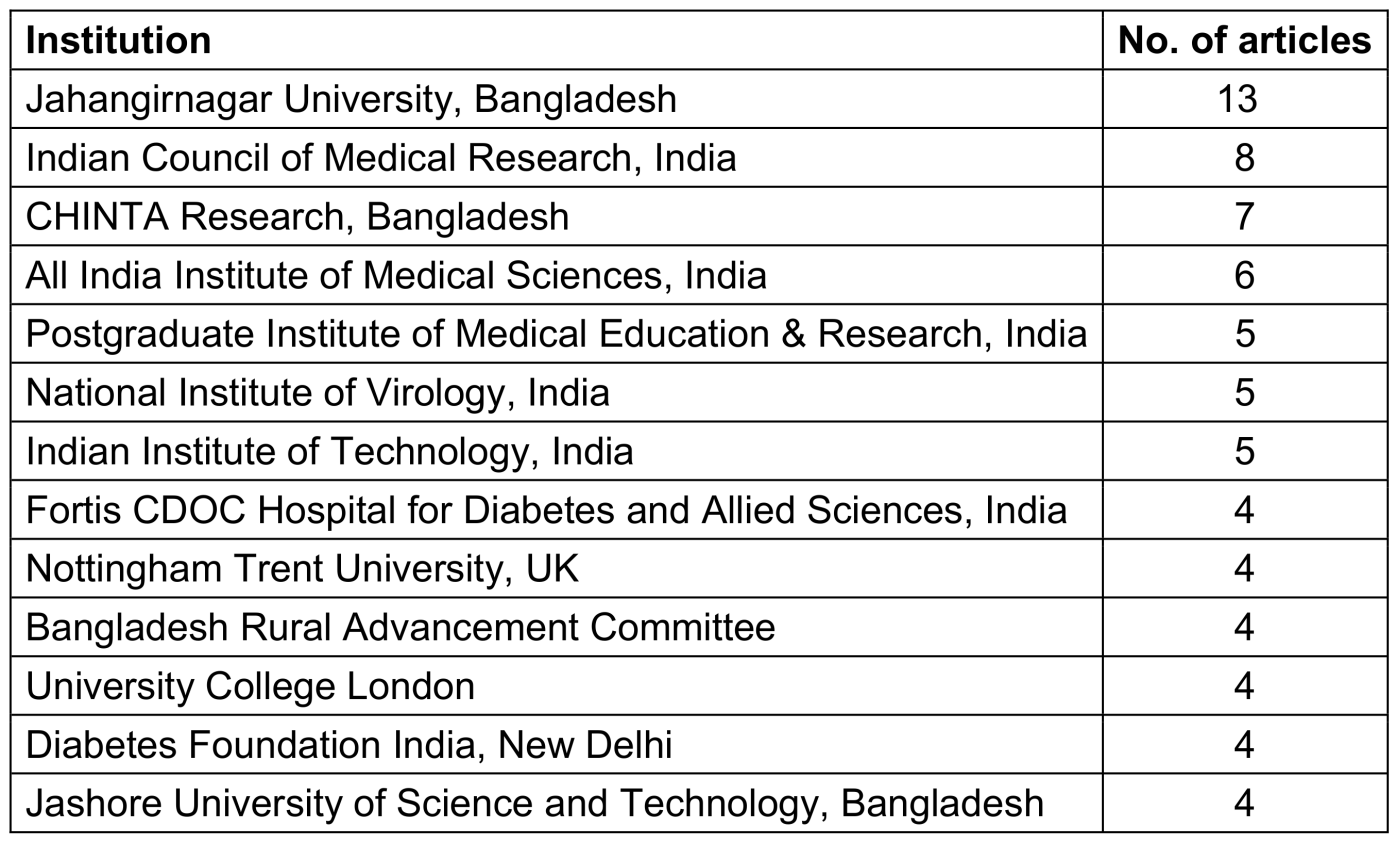
Institutes affiliated with 4 or more research publications

**Table 5:**
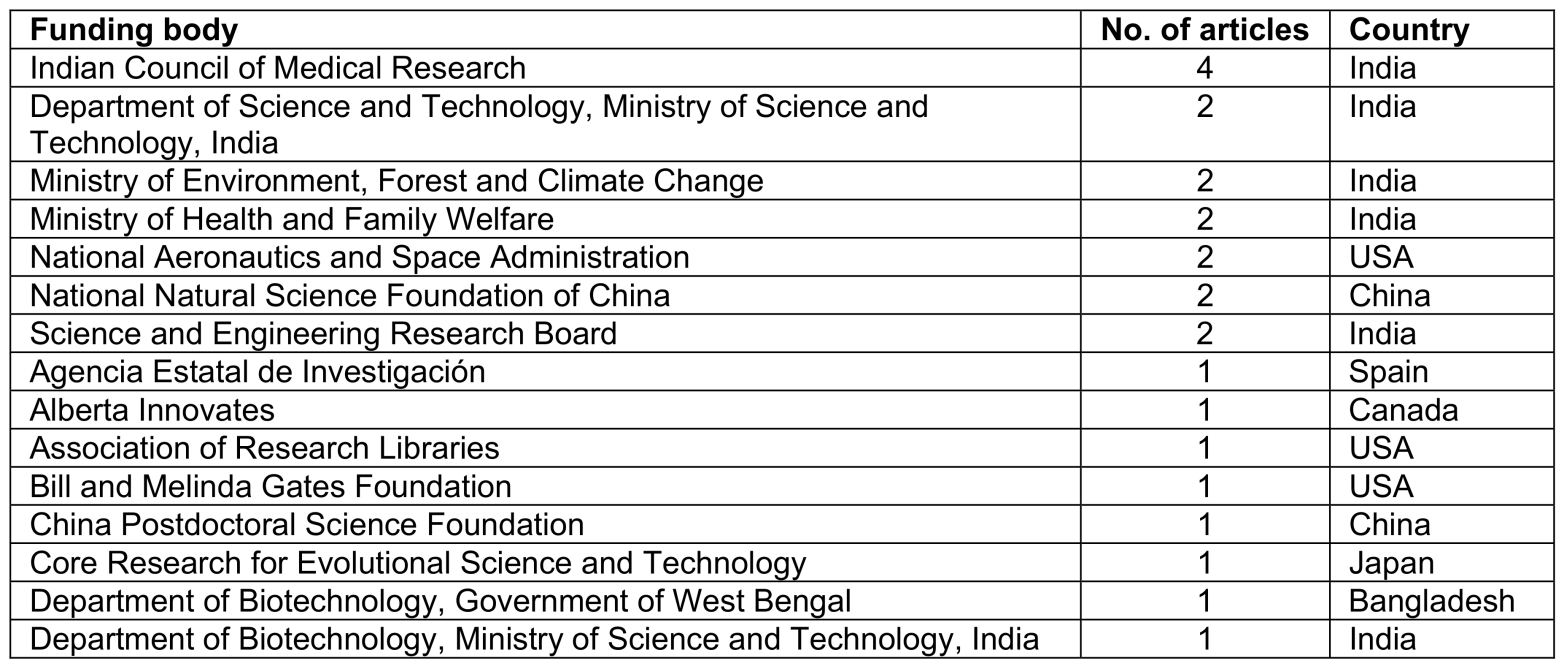
Top 15 funding bodies that supported COVID-19-related articles in South Asia

**Table 6:**
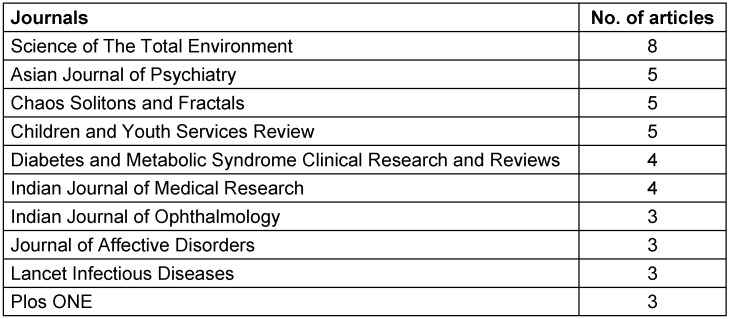
Journals with 3 or more articles published

**Table 7:**
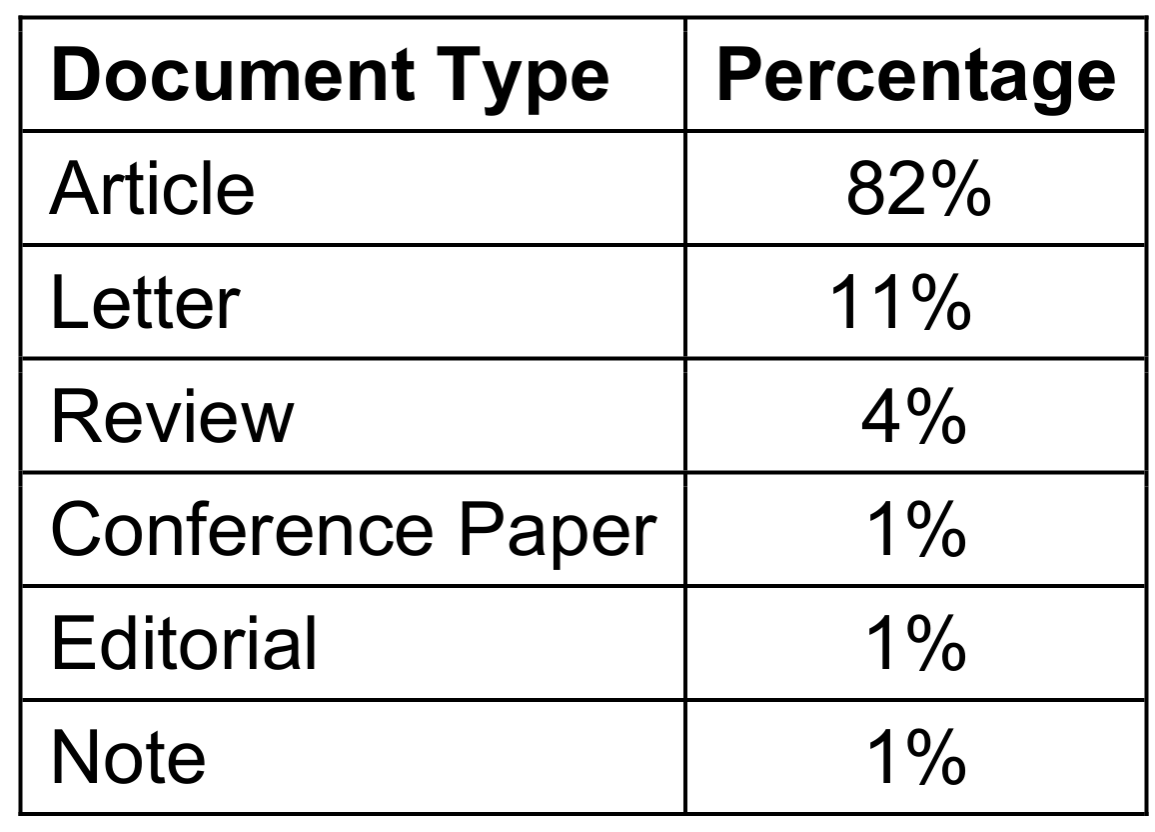
Document Type
